# Digitized Construction of Iontronic Pressure Sensor with Self-Defined Configuration and Widely Regulated Performance

**DOI:** 10.3390/s22166136

**Published:** 2022-08-16

**Authors:** Honghao Wang, Chun Liang, Haozhe Zhang, Yan Diao, Hua Luo, Yangyang Han, Xiaodong Wu

**Affiliations:** 1 School of Mechanical Engineering, Sichuan University, Chengdu 610065, China; 2 State Key Laboratory of Polymer Materials Engineering, Sichuan University, Chengdu 610065, China

**Keywords:** digitized fabrication, pressure sensor, iontronic sensing, physiological activity monitoring, electronic skin

## Abstract

Flexible pressure sensors are essential components for wearable smart devices and intelligent systems. Significant progress has been made in this area, reporting on excellent sensor performance and fascinating sensor functionalities. Nevertheless, geometrical and morphological engineering of pressure sensors is usually neglected, which, however, is significant for practical application. Here, we present a digitized manufacturing methodology to construct a new class of iontronic pressure sensors with optionally defined configurations and widely modulated performance. These pressure sensors are composed of self-defined electrode patterns prepared by a screen printing method and highly tunable pressure-sensitive microstructures fabricated using 3D printed templates. Importantly, the iontronic pressure sensors employ an iontronic capacitive sensing mechanism based on mechanically regulating the electrical double layer at the electrolyte/electrode interfaces. The resultant pressure sensors exhibit high sensitivity (58 kPa^−1^), fast response/recovery time (45 ms/75 ms), low detectability (6.64 Pa), and good repeatability (2000 cycles). Moreover, our pressure sensors show remarkable tunability and adaptability in device configuration and performance, which is challenging to achieve via conventional manufacturing processes. The promising applications of these iontronic pressure sensors in monitoring various human physiological activities, fabricating flexible electronic skin, and resolving the force variation during manipulation of an object with a robotic hand are successfully demonstrated.

## 1. Introduction

As an essential branch of wearable electronics and devices, flexible pressure sensors have attracted tremendous attention from both academic and industry areas, with extensive applications in wearable health monitoring and disease diagnosis, human–machine interfacing, artificial intelligent robotics, the Internet of Things, and other emerging fields [1,2,3,4,5,6,7,8,9,10]. In the past decade, significant progress has been made based on novel nanomaterials [11,12,13], unique microstructures [14,15,16], and new transduction mechanisms [17,18,19]. For the currently developed flexible pressure sensors, two basic components are generally indispensable. The first essential component is the flexible pressure sensing layer that can transduce the externally applied mechanical stimulations into a variation of specific electrical signals (e.g., resistance, capacitance, current, potential difference, etc.). The other necessary component is the flexible electrode pattern, which is used to acquire and transmit the responsive electrical signals generated by the pressure sensing layers. Flexible pressure sensors with specific device configurations can be assembled and constructed from the aforementioned pressure sensing layer and the flexible electrodes.

The construction of flexible pressure sensors generally involves the preparation of flexible electrodes, as well as the fabrication of pressure sensing layers. The flexible electrodes of pressure sensors are usually prepared by defined patterning of conductive materials (e.g., metals, conducting nanomaterials, conductive composites, etc.) via photolithography, chemical or physical vapor deposition, spray coating with shadow mask, ink-jet printing, and so on [20,21]. The key point for this process is how to form specific electrode patterns with the selected conductive materials in a controllable manner. For the flexible pressure sensing layers, they are usually prepared based on porous and compressible materials (e.g., aerogels, foams, fabrics, etc.) [22,23,24], or fabricated by creating microstructures on the surface of elastomeric materials using specific templates such as etched silicon wafers, abrasive papers, textiles, plant leaves, and so on [25,26,27]. By assembling the above-mentioned flexible electrodes and the pressure sensing layers, different categories of flexible pressure sensors with desirable performance (e.g., high sensitivity, low detection limit, good response linearity, etc.) [28,29,30] or unique features (e.g., biocompatibility, self-healing capability, good stretchability, etc.) [31,32,33] can be constructed.

Despite of the remarkable achievements made in the area of flexible pressure sensors, the manufacturing processes of these sensors generally lack good controllability and high tunability. More specifically, the geometry patterns, structural parameters, sensor configurations, and responsive properties of the developed pressure sensors are difficult to be widely modulated, which greatly limits their practical application scenarios. Optionally geometrical engineering and morphological tuning of flexible pressure sensors will be highly desired for manufacturing the next generation of wearable intelligent devices and systems, which, however, still remains a great challenge so far.

Digital manufacturing is a new discipline of manufacturing science that involves digital design, rapid prototyping, and digital management based on the customer demand and quality standards. With the support of design software, computer networks and additive manufacturing technologies, digital manufacturing enables the reduction of resource waste, improves product quality, shortens manufacturing time, and expand the product diversity. More importantly, digital manufacturing allows the product parameters to change and adjust flexibly, rapidly, and efficiently, which is difficult to realize via conventional manufacturing methodologies. With the further development and extension of digital manufacturing in more areas, digitized manufacturing of wearable electronics will be a trend in the future. Especially for wearable pressure sensors with specific electrode patterns and pressure sensing microstructures, fully digitized construction of pressure sensors can dramatically broaden their application range. Some digital manufacturing techniques (e.g., ink-jet printing, three-dimensional printing, laser processing, etc.) [34,35,36] have been involved in preparing the partial components of flexible pressure sensors. However, fully digitized design and construction of the entire pressure sensors with optionally defined configurations and widely tunable performance have rarely been explored to the best of our knowledge.

In this work, we propose and demonstrate a fully digitized approach to construct a new kind of iontronic flexible pressure sensor via the alliance of 2D printing and 3D printing methods. Specifically, flexible electrodes of pressure sensors with optionally defined geometric patterns are prepared by screen printing of a conductive ink on flexible polyethylene terephthalate (PET) substrate. Pressure-sensitive microstructures of the pressure sensors with highly tunable morphological features are fabricated using a 3D printed template via stereolithography (SLA). Based on these two digitized 2D and 3D printing methods, flexible pressure sensors with arbitrary geometrical patterns and morphological microstructures can be constructed facilely, rapidly, and efficiently. Furthermore, our pressure sensors utilize an iontronic capacitive sensing mechanism based on mechanically regulating the electrical double layer at the electrolyte/electrode interfaces with exceptionally high capacitance, which greatly improves the sensor sensitivity. Benefitting from this novel sensing mechanism and the digitized construction processes, the as-prepared pressure sensors exhibit high sensitivity (58 kPa^−1^), fast response and recovery time (45 ms and 75 ms), low detectability (6.64 Pa), and good repeatability (over 2000 cycles). More importantly, our pressure sensors show remarkable tunability and adaptability in sensor configuration and sensor performance, which is challenging to achieve via the conventional manufacturing processes. As proof-of-concept demonstrations, these pressure sensors are well qualified for monitoring various physiological activities of the human body (e.g., air blowing, pulse wave, heart rate, finger touch, etc.), and can also be used to fabricate pressure-sensitive electronic skins and smart robotic hands that can perceive the force variation when manipulating an object. This proposed digitized construction of iontronic flexible pressure sensors with superior tunability, adaptability, and sensor performance opens up new opportunities for the future manufacturing of smart wearables, intelligent robotics, and human–machine interfaces.

## 2. Results and Discussion

### 2.1. Digitized Construction of Flexible Iontronic Pressure Sensors

**Figure 1:**
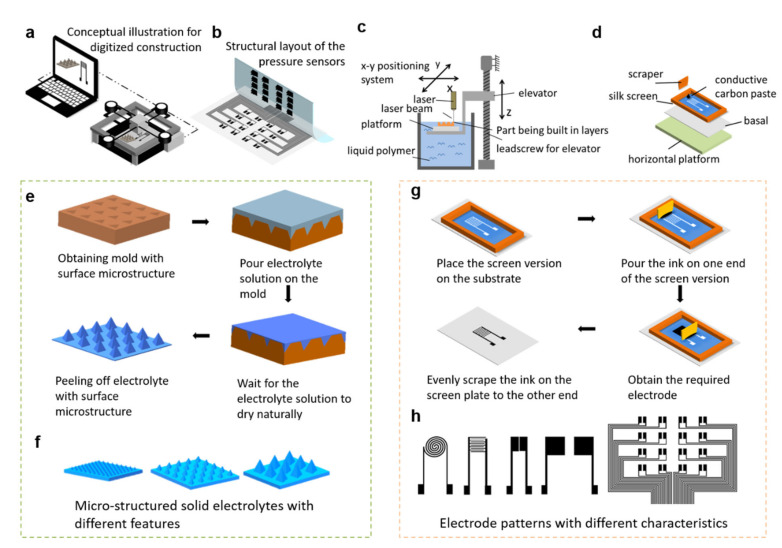
Design concept for the digitized fabrication of iontronic pressure sensors. (**a**) Conceptual schematic illustrating the digital manufacturing of iontronic pressure sensors based on 2D and 3D printing. (**b**) The device configuration of the pressure sensors. (**c**) Schematic showing the fabrication of the templates used to construct the micro-structured solid electrolyte based on SLA 3D printing. (**d**) Schematics depicting the basic configurations for the screen printing of electrode patterns. (**e**) Preparation process of the micro-structured solid electrolytes via a solution casting method. (**f**) Illustrations showing that micro-structured solid electrolytes with different shapes and sizes can be obtained using the digitized fabrication method. (**g**) Schematics depicting the screen printing process of the electrode patterns. (**h**) Schematic electrode patterns of different geometric characteristics can be prepared by the screen printing method.

The overall design concept and fabrication approach of the iontronic pressure sensors are illustrated in Figure 1a. The iontronic pressure sensors are mainly composed of two parts: (1) ionic solid electrolytes with periodic microstructure on the surface and (2) printed conductive electrodes with specific geometry (Figure 1b). Notably, all of the components of these iontronic pressure sensors are constructed based on digitized manufacturing processes. Specifically, the ionic solid electrolytes with periodic microstructures are prepared using a 3D-printed template based on stereolithography (SLA). The morphological parameters of the microstructures (including shape, size, spacing, density, etc.) can be defined easily via a 3D modeling software (e.g., Auto CAD) and implemented efficiently by the SLA 3D printing technique (Figure 1c). After obtaining the template, periodic microstructures can be readily created on the ionic solid electrolyte surface via a simple solution casting method (Figure 1e, more details are given in the Experimental Section). Using this process, ionic solid electrolytes with arbitrary microstructure characteristics can be digitally designed and constructed, as illustrated in Figure 1f. In addition to the micro-structured pressure sensing layer, the conductive electrode patterns can also be fabricated via digitized printing methods (Figure 1d). In this work, screen printing is selected due to its superior cost-efficiency, scalability, and flexibility in creating self-defined patterns when compared with other digital printing methods. Conductive electrode pattens with optional shapes and sizes can be designed via a 2D drawing software, and then can be printed onto a flexible substrate (e.g., PET film is used in this work) by the screen printing process (Figure 1g,h). Via the alliance of digitized 3D printing and 2D printing methods, flexible iontronic pressure sensors with arbitrary pressure sensing microstructure and conductive electrode patterns can be constructed. The performance of the iontronic pressure sensors can also be easily and widely regulated by tuning the microstructure features and electrode configurations, exhibiting superior flexibility and adaptability compared with the conventional pressure sensors based on non-digital fabrication methods.

### 2.2. Fabrication of Iontronic Pressure Sensors with Micro-Structured Solid Electrolytes

**Figure 2:**
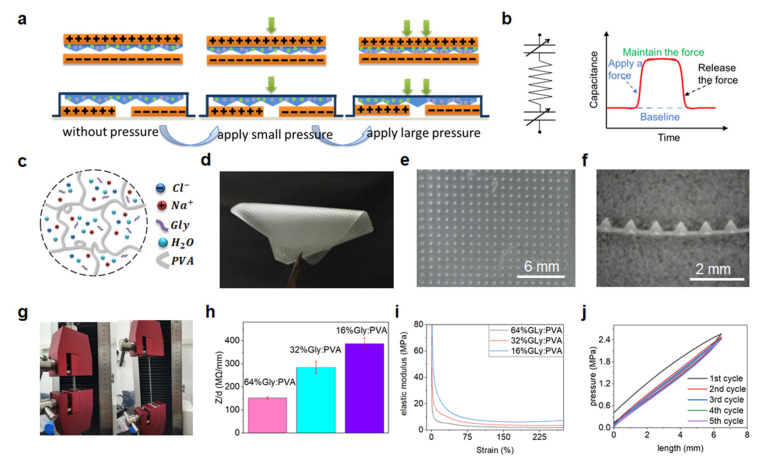
Construction of micro-structured solid electrolytes and the working principle of the iontronic pressure sensors. (**a**,**b**) Schematics showing the structural changes (including top-bottom electrode configuration and side-by-side electrode configuration), working principle (i.e., mechanical regulation of the EDL capacitance at the two electrolyte/electrode interfaces), and response behavior of the iontronic pressure sensors. (**c**,**d**) Schematic composition and digital picture of the PVA/NaCl/Gly/water ionic electrolytes with microstructures on the surface. (**e**,**f**) Optical microscope images showing the microstructure morphology created on the electrolyte surface, with uniform and periodic microstructures observed clearly. (**g**) Photographs showing the elasticity and stretchability of the PVA/NaCl/Gly/water ionic electrolytes. (**h**) Ionic impedance of PVA/NaCl/Gly/water electrolytes with different Gly/PVA ratios (16%, 32%, and 64%, respectively). (**i**) Stress-strain curves of PVA/NaCl/Gly/water electrolytes with different Gly/PVA ratios. (**j**) Mechanical cyclic test of the PVA/NaCl/Gly/water electrolyte with the Gly/PVA weight ratio of 32%.

The operating principle of the iontronic pressure sensors is based on the mechanical regulation of a unique interfacial electronic–ionic capacitance at the electrolyte/electrode interfaces, as illustrated in Figure 2a. Specifically, a micro-structured solid electrolyte with mobile ions is placed in direct contact with two flexible electrodes. The micro-structured electrolyte with massive cations and anions can form a unique supercapacitive layer with exceptionally high unit area capacitance at the electrolyte/electrode interface, which is usually called the electrical double layer (EDL). Such EDL capacitance at the electrolyte/electrode interface is proven to be more than 1000 times higher than that of the traditional parallel-plate devices [18]. Regulating the remarkable EDL capacitance with externally applied mechanical stimulations enables fabrication of mechanical sensors with much-improved sensitivity. Here, periodic and uniform microstructures with well-defined features are created on the surface of the ionic electrolyte, which allows us to continuously modulate the EDL capacitance at the electrolyte/electrode interface, as illustrated in Figure 2b. The electronic–ionic contact area between the micro-structured electrolyte and the electrodes is very small without externally applied pressure. A relatively low capacitance is measured between the two electrodes. With a pressure applied upon the device, the microstructures on the electrolyte surface are compressed and the electronic–ionic contact increases accordingly, resulting in a dramatic variation in the EDL capacitance. Such mechanical regulation of the EDL capacitance enables us to detect and monitor the externally applied force or pressure.

The micro-structured solid electrolytes are composed of polyvinyl alcohol (PVA), sodium chloride (NaCl), glycerol (Gly) and water, as illustrated in Figure 2c. PVA is used as the polymer matrix for the electrolytes, which endows the ionic electrolytes with shape-maintaining capability and good elasticity. NaCl is employed as the ion source, providing abundant mobile cations and anions within the electrolytes. Gly, which acts as a humectant that can bind with water molecules tightly, is used to adjust as well as preserve the water in the electrolytes. The properties of the solid electrolytes (including the water content, mechanical softness, and ionic conductivity) can be easily modulated by adjusting the Gly content. Here, micro-structured solid electrolytes with different Gly:PVA weight ratios (i.e., 16%, 32%, and 64%, respectively) are prepared (Figure 2d). The microstructures created on the solid electrolytes with high structural fidelity and good uniformity can be clearly observed from the optical microscope images (Figure 2e,f). The structural parameters (including the shape, size, spacing, density, and so on) can be easily and widely modulated based on the proposed digitized fabrication method in this work.

The prepared PVA/NaCl/Gly/water ionic electrolytes exhibit good elasticity and resilience, as presented in Figure 2g. The electrical and mechanical properties of the ionic electrolytes can also be facilely regulated by changing the Gly content. As shown in Figure 2h, ionic electrolytes with higher Gly content exhibit lower ionic impedance. This is due to the fact that higher Gly content gives rise to more water molecules in the electrolyte, improving both the activity and mobility of the ions. In addition, the Gly content also affects the mechanical properties of the ionic electrolytes. As indicated in Figure 2i, ionic electrolytes with higher Gly content show lower Young’s modulus and higher softness, which also arises from higher water content in the electrolytes. Notably, the prepared PVA/NaCl/Gly/water ionic electrolytes exhibit good elasticity, with low mechanical hysteresis measured in the mechanical cycling test (Figure 2j), which can endow the resultant iontronic pressure sensors with desirable stability and reproducibility.

### 2.3. Performance Characterization of Iontronic Pressure Sensors

**Figure 3:**
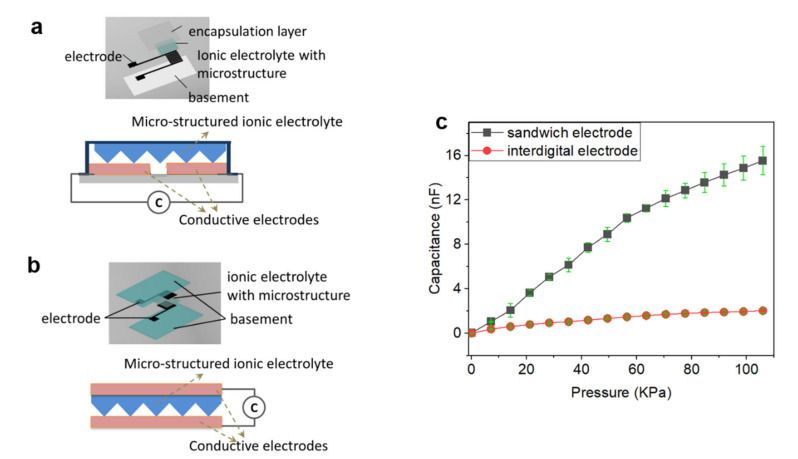
Iontronic pressure sensors with different device configurations. (**a**,**b**) The basic structure, components, and measurement of pressure sensors with different configurations: interdigital electrode configuration (**a**) and sandwich electrode structure (**b**). (**c**) Response behaviors of the pressure sensors with different device configurations.

Iontronic pressure sensors with two different device configurations are fabricated and their sensing performance is investigated, as given in Figure 3a–c and discussed in Figure S1. Figure 3a shows the pressure sensors with an interdigital electrode configuration. A micro-structured solid electrolyte is placed on the top of the two interdigitated electrodes in the same plane. In comparison, pressure sensors with a sandwich electrode configuration are presented in Figure 3b, with a micro-structured solid electrolyte sandwiched between the top and bottom electrodes in different planes. Compared with the pressure sensors with interdigital electrode configuration, the pressure sensors with sandwich electrode configuration exhibit relatively higher sensitivity (Figure 3c). Nevertheless, pressure sensors with interdigital electrode configuration are more compact and more convenient to use in practical applications. In addition, the relatively low sensitivity of pressure sensors with interdigital electrode configuration can be compensated and easily improved by other strategies (e.g., component or structural regulations of the electrolytes as demonstrated below). Hence, pressure sensors with interdigital electrode configuration are employed for the performance evaluations and application demonstrations unless otherwise specified.

**Figure 4:**
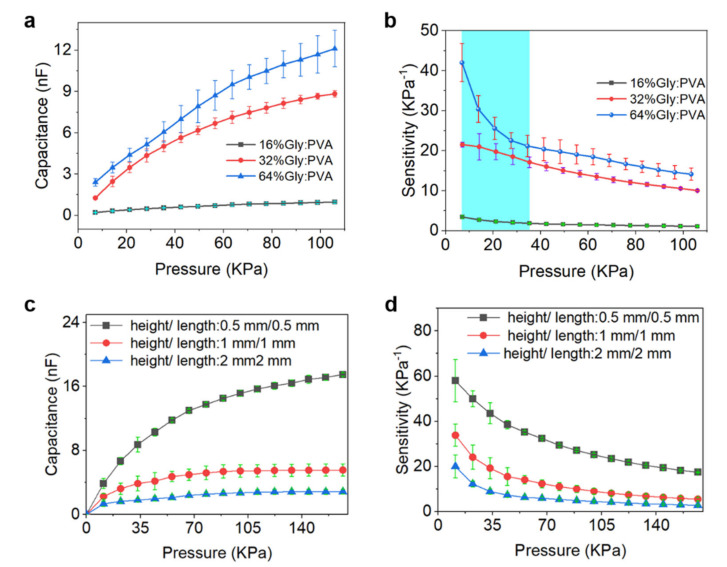
Component regulation and structural regulation of the iontronic pressure sensors. (**a**,**b**) Capacitance variations (**a**) and sensitivity variations (**b**) of the iontronic pressure sensors fabricated with electrolytes of different Gly/PVA ratios (16%, 32%, and 64%, respectively). Electrolyte of 32% Gly:PVA ratio are used to construct the sensors in the following measurements unless otherwise specified. (**c**,**d**) Capacitance variations (**c**) and sensitivity variations (**d**) of the iontronic pressure sensors fabricated using electrolytes with different geometrical parameters (the height/length of the micro-tetrahedrons is 0.5 mm, 1 mm, and 2 mm, respectively).

The sensing performance of the iontronic pressure sensors can be widely tuned and adjusted via component regulation of the ionic electrolytes. As shown in Figure 4a, pressure sensors fabricated with micro-structured electrolytes of different Gly:PVA weight ratios exhibit remarkable difference in the sensing performance. Specifically, pressure sensors with electrolytes of 64% and 32% Gly:PVA ratios exhibit much higher capacitance variation than that of sensors with electrolytes of 16% Gly:PVA ratio under the same applied pressures. Here, the sensor sensitivity can be defined as (C − C_0_)/P, namely, the relative capacitance change (C − C_0_) caused by the externally applied pressure (P). As presented in Figure 4b, pressure sensors with electrolytes of 64% Gly:PVA ratio and 32% Gly:PVA ratio show a high sensitivity of 42 kPa^−1^ and 21 kPa^−1^, respectively, under the pressure of 5 kPa, which is much higher than that of sensors with electrolytes of 16% Gly:PVA ratio (3.4 kPa^−1^). This can be attributed to the water absorption and preservation effects of the humectant Gly that is incorporated into the solid electrolytes. High Gly content gives rise to high water content of the electrolytes, resulting in higher ion mobility and better softness of the solid electrolytes. Therefore, the sensing performance of the iontronic pressure sensors can be highly tunable via component regulation of the electrolytes.

On the other hand, the sensing performance of the iontronic pressure sensors can also be efficiently modulated via structural regulation of the electrolytes (i.e., tuning the size and density of the microstructures on the electrolyte surface). Typically, micro-tetrahedrons are created on the electrolyte surface via the proposed SLA 3D printing method. The effect of the morphological parameters of the micro-tetrahedrons on the sensor performance is investigated. Micro-tetrahedrons with different heights and lengths (i.e., 0.5 mm, 1 mm, and 2 mm, respectively) are created, and the corresponding sensor performance is given in Figure 4c. It is noted that sensors fabricated with micro-tetrahedrons with the heights and lengths of 0.5 mm exhibit the highest sensitivity (58 kPa^−1^), which is much higher than the sensitivity (20 kPa^−1^) of sensors fabricated with micro-tetrahedrons with the heights and lengths of 2 mm. This can be attributed to the fact that smaller micro-tetrahedrons are easier to be deformed by the externally applied pressure than bigger micro-tetrahedrons, thus resulting in larger variation in the electrolyte/electrode contact area. This result reveals that the sensing performance of the iontronic pressure sensors can also be modulated via structural regulation of the electrolytes.

**Figure 5:**
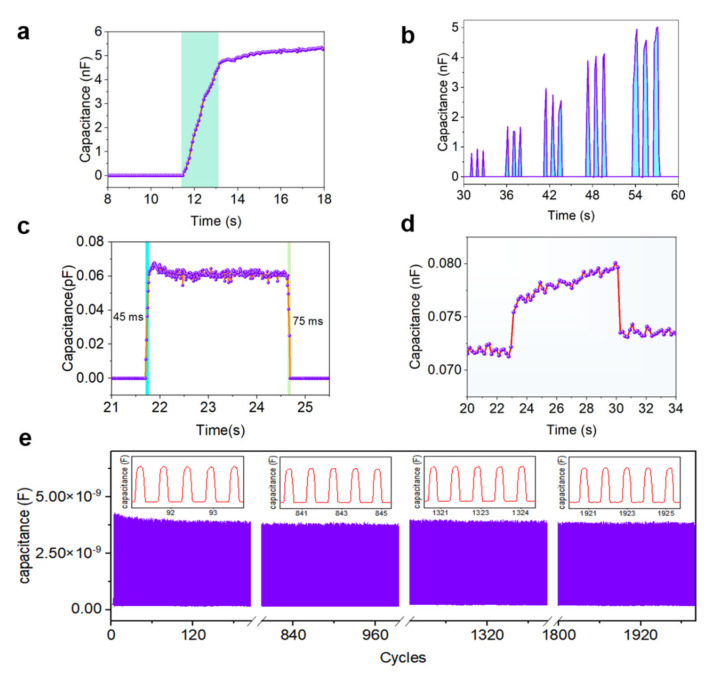
General properties of the iontronic pressure sensors based on digitized construction. (**a**) Typical response behavior of the sensors with a pressure of ≈32 kPa gradually applied upon the device. (**b**) Response curve of the pressure sensors when pressures with different intensities are applied on the device repeatedly for three times. The pressure intensities are about 4.8 kPa, 9.1 kPa, 16.0 kPa, 24.4 kPa and 33.4 kPa, respectively. (**c**) Response time and recovery time characterization of the prepared iontronic pressure sensors measured with an applied pressure of ≈3.5 kPa. (**d**) Response behavior of the sensors when a tiny pressure of 6.64 Pa is loaded and unloaded on the device. (**e**) Reliability and reproducibility test of the iontronic pressure sensors with a pressure of 27 kPa loaded repeatedly on the device for 2000 cycles.

The typical responsive behaviors of the iontronic pressure sensors to externally applied mechanical stimulations are investigated and displayed in Figure 5. When applying a pressure gradually upon the sensors, the measured capacitance signal shows a continuous and gradual increase, and then stays relatively stable when the pressure is held (Figure 5a). In addition, when applying a varying pressure with different intensities on the device repeatedly, the variation process of the applied pressure can be monitored in real time (Figure 5b). These results confirm the good capability of the iontronic pressure sensors for continuously monitoring the pressure variations. The response time and recovery time of the pressure sensors are measured to be 45 ms and 75 ms, respectively (Figure 5c), which are fast enough to monitor a diversity of human physiological activities in practical applications. In addition, when a subtle pressure of 6.64 Pa is applied upon and removed from the pressure sensors, an obvious variation in the sensor response signal can be distinguished (Figure 5d), revealing a low detectability of such iontronic pressure sensors.

In addition, the mechanical cyclic test is conducted to evaluate the reliability and reproducibility of the iontronic pressure sensors. As shown in Figure 5e, the response signals of the pressure sensors show a slight variation in the beginning tens of cycles, and then become highly stable subsequently during the remaining cyclic test. The signal patterns of the sensors generated in the first five cycles and last five cycles are very similar to each other, demonstrating the good reproducibility and reliability of the pressure sensors. All of these results mentioned above indicate that the proposed iontronic pressure sensors have comparable sensor performance in the terms of sensitivity, response and recovery speed, detectability, and reliability when compared with other pressure sensors reported in the literature (Table S1). Importantly, our iontronic sensors have obvious superiority in terms of the sensor tunability, controllability, and adaptability enabled by the proposed digitized manufacturing methodology, exhibiting broad application prospects in the future design and fabrication of smart wearable devices and systems.

### 2.4. Application of Iontronic Pressure Sensors in the Monitoring of Physiological Activities

**Figure 6:**
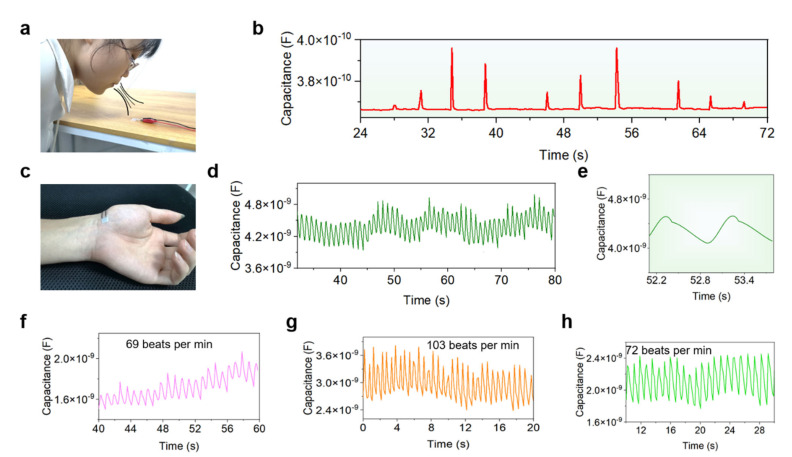
Monitoring of physiological signals and activities based on the flexible iontronic pressure sensors. (**a**) Digital picture showing that a subject is blowing air of different intensities towards a sensor. (**b**) Response signals of the sensor to airflows with different intensities, indicating high sensitivity of the sensor. (**c**) Photograph showing a sensor mounted on the wrist of a subject for pulse signal monitoring. (**d**,**e**) Continuously recorded pulse signals (**d**) and enlarged pulse waveforms (**e**) of the subject with the iontronic pressure sensor. (**f**–**h**) Pulse signals recorded at different physiological status, including before exercise (**f**) and one minute after exercise (**g**), and five minutes after exercise (**h**).

Benefitting from the high sensitivity, remarkable tunability, and adaptability of the iontronic pressure sensors enabled by the newly proposed digitized construction methodology, a diversity of human physiological activities can be monitored and analyzed with such sensors. Firstly, we examined the capability of the sensors for detecting the weak airflows generated by blowing air from a subject’s mouth. As shown in Figure 6a, an iontronic pressure sensor is attached on a stable table and the subject blows air towards the sensor. Different types (e.g., duration, intensity, etc.) of air blows cause different air pressures applied on the sensor, which gives rise to different signal outputs of the sensors (Figure 6b). Specifically, when blowing air onto the sensor, there is an immediate uprising in the signal outputs, which then go back to the initial value when the air blow stops. Notably, with the increase of the blowing intensity, signal patterns with different magnitudes can be observed clearly, indicating good capability of the sensors in detecting airflow. Moreover, when increasing and then decreasing the airflow repeatedly, the sensor signal outputs exhibit a similar variation trend, revealing the good reliability of the sensors.

In addition, a flexible pressure sensor is attached to the wrist of a subject to record the artery pulse signals in real time, as presented in Figure 6c. Continuous pulse waveforms can be acquired with this iontronic pressure sensor (Figure 6d). Generally, the pulse signals are relatively stable, despite of some fluctuation in the baseline. A heart rate of 69 beats per minute for this subject can be calculated from the recorded pulse signals. In addition, two typical pulse waveforms are extracted and amplified for clear observation, as depicted in Figure 6e. It is found that two distinguishable peaks (a high peak followed by a low peak) can be distinguished on the graph, which is consistent with the heartbeat activity of the human body.

To further investigate the sensor’s capability for detecting the heart rate at different physiological conditions, the pulse signals of the subject before exercise, one minute after exercise, and five minutes after exercise are recorded and analyzed, respectively, as shown in Figure 6f–h and Figure S2. Figure 6f shows the heart rate of the subject at rest status before taking exercise (i.e., 69 beats per minute), which is in good accordance with the normal heart rate range (i.e., 60–100 beats per minute) for healthy people. In contrast, after taking a short period of exercise (i.e., climbing four flights of stairs continuously), the heart rate of the subject increases up to 103 beats per minute, as presented in Figure 6g. This is because performing vigorous exercise can rapidly elevate the heart rate, which is a very normal physiological phenomena for human body. After taking a short break after exercise (i.e., have 5 min rest), the elevated heart rate goes back to a normal state, which is measured to be 72 beats per minute (Figure 6h). The whole process of the heart rate variation fits the actual situation very well, indicating the good capability of our sensors in detecting the heart rate of the human body.

### 2.5. Application of Iontronic Pressure Sensors for Fabricating Pressure-Sensitive Electronic Skins

**Figure 7:**
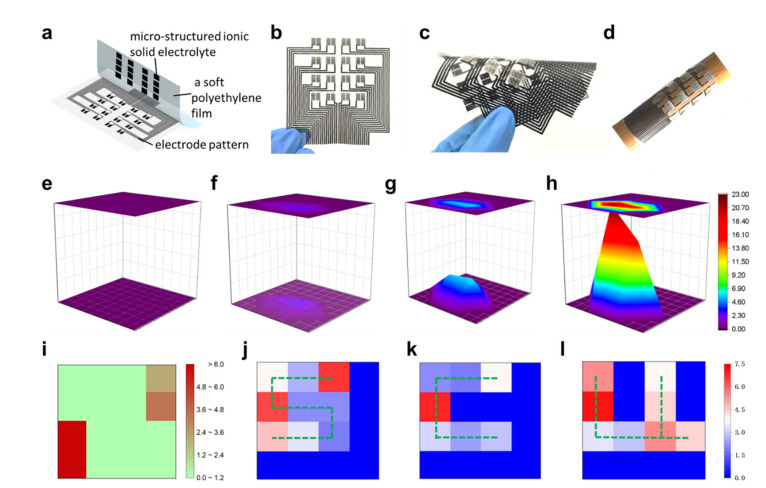
Pressure-sensitive electronic skin for detecting pressure magnitude and pressure distribution. (**a**) Schematic depicting the device structure and electrode configuration of the pressure-sensitive electronic skin. (**b**) Digital picture of the fabricated pressure-sensitive electronic skin. (**c**,**d**) Digital photographs showing the pressure-sensitive electronic skin in different bending status, revealing good flexibility of the electronic skin. (**e**–**h**) Spatial mappings of capacitance signal variations when a single-point pressure with different magnitudes (0 kPa, 28.5 kPa, 63.3 kPa and 143.8 kPa, respectively) is applied on the electronic skin. (**i**) Spatial mappings of capacitance signal variations when two pressures of different magnitudes are applied on the diagonal corners of the electronic skin. (**j**–**l**) Spatial mappings of capacitance signal variations when objects with the letter shapes of “S”, “C”, and “U” are pressed onto the electronic skin. From the detected pressure distributions, the “S”, “C”, and “U” letter shapes can be recognized.

In addition to detecting human physiological signals, the iontronic pressure sensors can also be used to resolve the pressure magnitude and the pressure distribution. As a demonstration, a pressure-sensitive electronic skin with a 4 × 4 sensor array is designed and fabricated. As shown in Figure 7a and Figure S3, electrode patterns with a side-by-side electrode configuration are prepared by the screen printing technique. An array of micro-structured ionic solid electrolytes is placed on the sensing areas of the printed electrode patterns. After encapsulation, a flexible pressure-sensitive electronic skin is constructed, as shown in Figure 7b. This electronic skin can be bent into different status (Figure 7c) and even wrapped onto a wooden stick (Figure 7d), showing high flexibility and deformability.

To evaluate the capability of the electronic skin in detecting the pressure magnitude, a single-point pressure with different magnitudes is applied on the electronic skin. Without pressure applied on the electronic skin, the recorded capacitance signals of different sensing units show no variations (Figure 7e). When a pressure is applied to a specific location on the electronic skin, the spatial mapping of capacitance signals shows obvious variations (Figure 7f–h). Notably, as the magnitude of the applied pressure increases, the capacitance signal variations in the pressure-applied area show higher intensity, exhibiting more dramatic change in the color contrast mapping. These results verify the good ability of the electronic skin to detect the magnitude of the externally applied pressure.

In addition, the feasibility of the electronic skin for detecting multi-point pressure variations is explored. Firstly, a high pressure and a low pressure are applied onto the diagonal corners of the electronic skin. As shown in Figure 7i, the capacitance signal variations detected at the two diagonal corners are substantially different, which can be clearly reflected from the color contrast mapping. This indicates that the electronic skin is capable of detecting multi-point pressures. Furthermore, objects with the letter shapes of “S”, “C”, and “U” (i.e., the logo letters of our university) are pressed onto the electronic skin and the corresponding capacitance signal variations are recorded. As shown in Figure 7j–l, the pressure distributions in the letter shapes of “S”, “C”, and “U” can be clearly distinguished in the color contrast mapping, which further demonstrates the capability of the electronic skin for resolving the pressure distribution. Overall, the results above show that our electronic skin is well qualified for detecting both pressure magnitude and pressure distribution.

### 2.6. Application of Iontronic Pressure Sensors for Monitoring the Force Variation during Manipulation of an Object with a Robotic Hand

**Figure 8:**
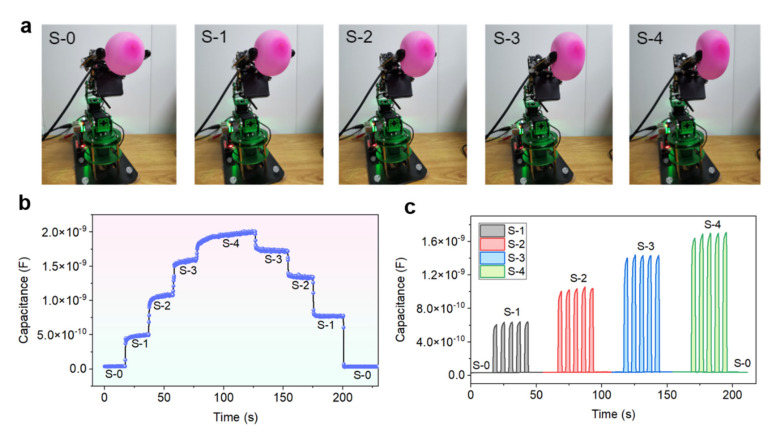
Monitoring of the force variation during manipulation of an object with a robotic hand with the flexible iontronic pressure sensors. (**a**) Digital pictures showing that a robotic hand grasps an object (e.g., a balloon is used here) with different forces. The grasping force increases gradually from the motion of S-0 to the motion of S-4. (**b**) Typical response signals of the flexible sensors during grasping and releasing of the object in stagewise manner, i.e., the motions of S-0 to S-4 were held for certain period of time before conducting the next motion. (**c**) Signal outputs of the sensors recorded when the robotic hand grasps the balloon repeatedly with different motions (S-1, S-2, S-3, and S-4).

Our body can perceive external mechanical stimuli easily through the mechanoreceptors located in the skin, which is vital for our survival. Tactile perception also plays an important role for intelligent robotics. However, endowing the smart robotics with mechanical perception capability is difficult to implement in practical applications. Here, as a demonstration, an iontronic pressure sensor is integrated onto one finger of a robotic hand to monitor the force variation during manipulation of an object. Figure 8a shows that the robotic hand manipulated a pink balloon in different statuses. With pressure sensors integrated on the finger, the manipulating force can be detected and recorded continuously, as given in Figure 8b. When the robotic hand grasps the balloon with four different magnitudes of forces, the balloon is deformed into different shapes, with stagewise sensor signal outputs observed. This indicates the good capability of the iontronic pressure sensors for continuously resolving the grasping force variations. In addition, the grasping motions with different force magnitudes are repeated for five cycles to evaluate the reliability of the sensors. As shown in Figure 8c, the signal output of the sensors show similar characteristics (e.g., height, width, etc.) when conducting the same grasping motion. When increasing the force magnitude of the grasping motions, the sensor output signals also increase correspondingly. These results reveal that the iontronic pressure sensors have good capability and reliability to detect and monitor the force variation during manipulation of an object with a robotic hand.

## 3. Conclusions

In summary, a digitized manufacturing methodology to construct a new kind of iontronic flexible pressure sensor is proposed and demonstrated in this work. Optionally defined electrode patterns can be prepared by the screen printing of a conductive ink on flexible PET substrate. Pressure-sensitive microstructures with highly tunable morphological features can be fabricated using SLA 3D printed templates. By assembling the printed electrode patterns and the pressure-sensitive microstructures, flexible iontronic pressure sensors with optional geometrical and morphological features can be constructed facilely, rapidly, and efficiently. The as-prepared pressure sensors exhibit high sensitivity (58 kPa^−1^), fast response and recovery time (45 ms and 75 ms), low detectability (6.64 Pa), good repeatability (over 2000 cycles), and more importantly, superior tunability and adaptability in sensor configuration and sensor performance. These merits are challenging to achieve simultaneously via the conventional sensor fabrication methods. We also demonstrate that these iontronic pressure sensors can be used for monitoring various physiological activities of the human body. Furthermore, such iontronic pressure sensors can also be used to fabricate pressure-sensitive electronic skins and smart robotic hands that can perceive the force variation when manipulating an object. The proposed digitized sensor construction methodology, along with the superior comprehensive sensor performance, will find broad application prospects in wearable healthcare devices, human–machine interfaces, intelligent robots, and other emerging fields.

## 4. Experiment and Characterization

### 4.1. Preparation of Conductive Electrode Patterns

PET film (100 μm or 25 μm in thickness) was used as the flexible substrate for screenprinting of the electrode patterns with conductive carbon ink (CH-8-MOD2, Jujo Printing Supplies & Technology (Pinghu) Co. Tokyo, Japan). The electrode patterns with different geometrical features were designed with commonly used 2D design software or Microsoft Powerpoint. Screen meshes (Nylon, 200 mesh) were prepared according to the geometry design of the self-defined electrode patterns. The screen printing process was conducted manually on a custom-made screen printing setup. After finishing the printing, the electrode patterns were annealed at 100 °C for 20 min on a hotplate with controlled temperature.

### 4.2. Fabrication of Micro-Structured Ionic Solid Electrolytes

The pressure-sensitive microstructures with self-defined morphological features were designed by commonly used 3D design software. The template for constructing the pressure-sensitive microstructures was manufactured by a commercial SLA 3D printing service company (Shangran Design Co. LTD, Shenzhen, China). After obtaining the 3D printed template, aqueous solutions including 25% wt% PVA, 100 mM NaCl, and a certain amount of Gly (the mass ratios of Gly to PVA were 16%, 32%, and 64%, respectively) were degassed and poured on the printed template with self-defined microstructures. Then, the electrolyte solutions were dried at room temperature for 24 h. After drying, ionic solid electrolyte films with periodic and uniform microstructures on the surface can be peeled off from the template and cut into a specific shape and size for subsequent use.

### 4.3. Construction of Flexible Iontronic Pressure Sensors and Pressure-Sensitive Electronic Skin

Flexible iontronic pressure sensors were constructed by assembling the micro-structured ionic electrolytes and the printed conductive electrode patterns. To fabricate pressure sensors with interdigitated electrode configuration, the micro-structured electrolytes with the desired size were placed on the sensing area of the printed interdigitated electrodes, which were then encapsulated with a soft polyethylene film. To fabricate pressure sensors with sandwiched electrode configuration, the micro-structured electrolytes were sandwiched between two flexible tetragonal electrodes, which were then encapsulated with a soft polyethylene film. A pressure sensor array with 4 rows and 4 columns was designed to fabricate the pressure-sensitive electronic skin. The electrode patterns of the electronic skin were screen-printed using the conditions mentioned above. Sixteen pieces of micro-structured electrolytes with a 32% Gly:PVA ratio and micro-tetrahedrons with a height/length of 1 mm/1 mm were placed on the sensing areas of the electronic skin. The electronic skin was then encapsulated with a polyethylene film. The sizing information of the electronic skin is given in Figure S3.

### 4.4. Characterization and Measurement

The capacitance signals of the sensors were measured using a high-speed Inductance-Capacitance-Resistance (LCR) meter (TH2817B, China) or using a portable precision multi-channel capacitance tester (LinkZill 01RC, China). The optical microscope observation was conducted on an optical microscope (SN-300, China). The mechanical performance tests of the ionic solid electrolytes were carried out on a microcomputer electronic universal material testing machine (QLW-5E, China). The manual test rack (HANDPI -HLD, China) was employed in the stepwise pressure test of the sensors. A custom-made mechanical testing setup integrated with a digital force meter (HP-100, HANDPI), a sliding stage, and a programmable stepping motor (KH-01, China) was used for the force and pressure measurement in the experiments. All human physiological activities monitoring experiments performed on human subjects were carried out with informed consent under the approval of the Scientific Ethical Committee of the School of Mechanical Engineering, Sichuan University.

## Data Availability

Not applicable.

## Supplementary Materials

The following supporting information can be downloaded at: https://www.mdpi.com/article/10.3390/s22166136/s1, Table S1: Comparison of the characteristics and the sensitivity of our pressure sensors with other reported pressure sensors in literature.; Figure S1: Schematics showing the structural variations of the sandwich electrode configuration and the side-by-side electrode configuration under externally applied pressure.; Figure S2: The extracted heart rates from the original pulse signals at different physiological statuses.; Figure S3: Digital picture showing the electronic skin with 16 sensors, along with the size information.
