# Supplementary material for: Digitized Construction of Iontronic Pressure Sensor with Self-Defined Configuration and Widely Regulated Performance
Source: Sensors (Basel). 2022 Aug 16;22(16):6136. doi: 10.3390/s22166136 (PMC9415562; doi:10.3390/s22166136)
Supplement: Supplementary file 1 [file sensors-22-06136-s001.zip › sensors-1877164-supplementary.pdf]

Digitized Construction of Iontronic Pressure Sensor with Self-Defined Configuration and Widely-Regulated Performance

Honghao Wang[1#], Chun Liang[1#], Haozhe Zhang[1], Yan Diao[1], Hua Luo[1]*, Yangyang Han[2], and Xiaodong Wu[1]*

[1]*School of Mechanical Engineering, Sichuan University, Chengdu 610065, China*

[2] *State Key Laboratory of Polymer Materials Engineering, Sichuan University, Chengdu 610065, China*

[#]*These authors contributed equally to this work.*

*E-mail: luohua@scu.edu.cn; xiaodong_wu@scu.edu.cn

Table S1. Comparison of the characteristics and the sensitivity of our pressure sensors with other reported pressure sensors in literature.

No.	Characteristic	Sensitivity /kPa^{-1}	Ref.
1	bioinspired hierarchical structure	8.5	*Adv. Mater.* **2016**, *28*, 5300
2	surface-wrinkled pyramidal microstructure	0.7	*Adv. Mater.* **2018**, *30*, 1803388
3	biomimetic hierarchical structures	19.8	*Adv. Funct. Mater.* **2017**, *27*, 1606066
4	3D graphene–poly(dimethylsiloxane) (GP) hollow structure	15.9	*Adv. Mater*. **2017**, *29*, 1702675
5	bionic spinosum microstructure.	25.1	*ACS Nano* **2018**, *12*, 2346
6	Silk-Molded structure	1.8	*Adv. Mater*. **2014**, *26*, 1336
7	Interlocked microdome struture	15.1	*ACS Nano* **2014**, *8*, 4689
8	ordered microstructures	5.5	*Small* **2014**, *10*, 3625
9	Digitally fabricated pressure sensors	58	***This work***

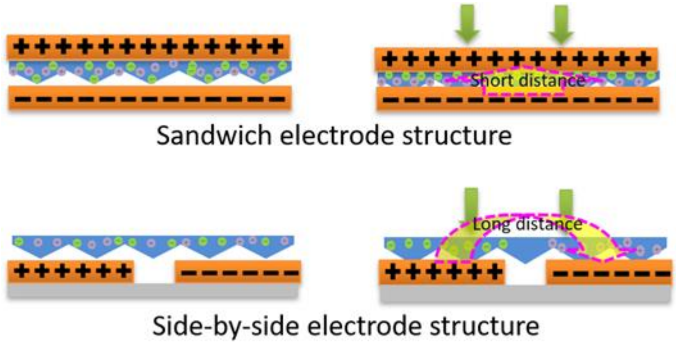

Figure S1. Schematics showing the structural variations of the sandwich electrode configuration and the side-by-side electrode configuration under externally applied pressure.

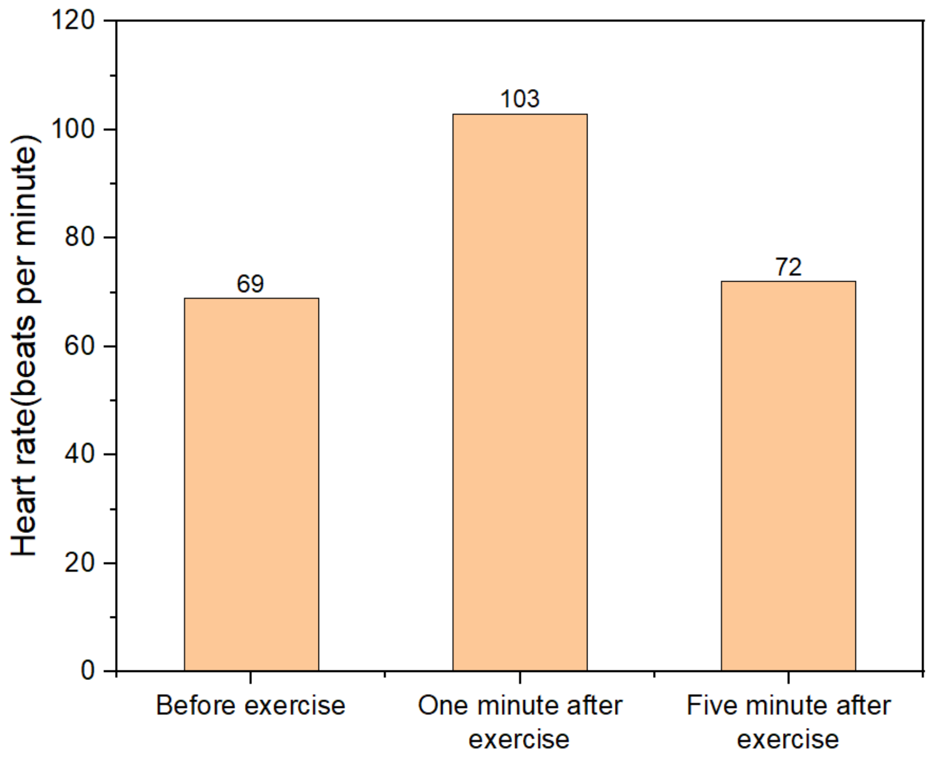

Figure S2. The extracted heart rates from the original pulse signals at different physiological statuses, including before exercise, one minute after exercise, and five minutes after exercise.

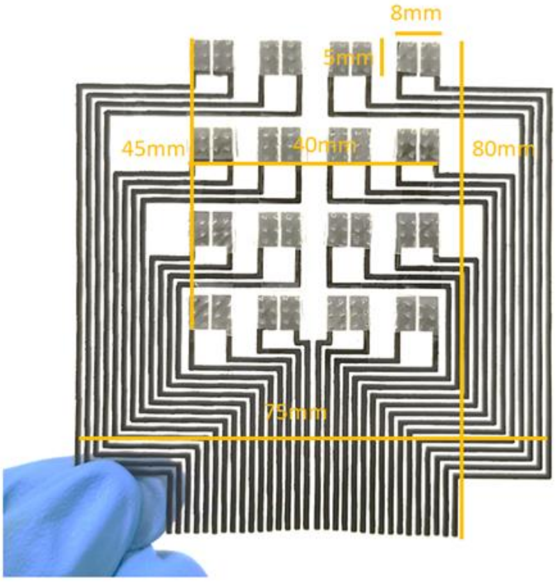

Figure S3. Digital picture showing the electronic skin with 16 sensors, along with the size information.
